# Chloroplast-localized GUN1 contributes to the acquisition of basal thermotolerance in *Arabidopsis thaliana*

**DOI:** 10.3389/fpls.2022.1058831

**Published:** 2022-12-22

**Authors:** Cecilia Lasorella, Stefania Fortunato, Nunzio Dipierro, Nicolaj Jeran, Luca Tadini, Federico Vita, Paolo Pesaresi, Maria Concetta de Pinto

**Affiliations:** ^1^ Department of Bioscience, Biotechnology and Environment University of Bari Aldo Moro, Bari, Italy; ^2^ Department of Biosciences, University of Milano, Milano, Italy

**Keywords:** heat stress, GENOMES UNCOUPLED 1, reactive oxygen species, redox regulation, retrograde signaling, thermotolerance

## Abstract

Heat stress (HS) severely affects different cellular compartments operating in metabolic processes and represents a critical threat to plant growth and yield. Chloroplasts are crucial for heat stress response (HSR), signaling to the nucleus the environmental challenge and adjusting metabolic and biosynthetic functions accordingly. GENOMES UNCOUPLED 1 (GUN1), a chloroplast-localized protein, has been recognized as one of the main players of chloroplast retrograde signaling. Here, we investigate HSR in Arabidopsis wild-type and *gun1* plantlets subjected to 2 hours of HS at 45°C. In wild-type plants, Reactive Oxygen Species (ROS) accumulate promptly after HS, contributing to transiently oxidize the cellular environment and acting as signaling molecules. After 3 hours of physiological recovery at growth temperature (22°C), the induction of enzymatic and non-enzymatic antioxidants prevents oxidative damage. On the other hand, *gun1* mutants fail to induce the oxidative burst immediately after HS and accumulate ROS and oxidative damage after 3 hours of recovery at 22°C, thus resulting in enhanced sensitivity to HS. These data suggest that GUN1 is required to oxidize the cellular environment, participating in the acquisition of basal thermotolerance through the redox-dependent plastid-to-nucleus communication.

## Introduction

Plants are constantly exposed to abiotic stresses throughout their entire life cycle, which heavily impact growth and yield. The effects of climate change increase the frequency and intensity of extreme events such as heat waves, compromising plant development and crop productivity irreversibly (Bita and Gerats, 2013). Among abiotic stresses, heat stress (HS) is considered one of the most detrimental for plants, since extreme temperature fluctuations cause impairment in essential biochemical and physiological processes (Hasanuzzaman et al., 2013). As sessile organisms, plants sense and respond to adverse environmental conditions activating defense systems (Zhu, 2016). The study of the mechanisms involved in plant perception and response to heat has, therefore, a great relevance in the actual climatic scenario.

Considering that photosynthesis-related processes are sensitive to thermal fluctuations, chloroplasts have been proposed as sensors of HS (Sun and Guo, 2016). Among the chloroplast protein complexes, the photosystem II, its oxygen-evolving complex, the electron transport chain and the carbon fixation system are particularly prone to damage due to high temperatures (Allakhverdiev et al., 2008). Furthermore, heat stress reduces the content of photosynthesis-associated pigments and alters cell membrane stability by protein denaturation and lipid peroxidation (Wise et al., 2004; Wahid et al., 2007; Allakhverdiev et al., 2008). The HS-mediated damage to photosynthetic apparatus inhibits the excitation energy transfer and the electron transport in the chloroplast, leading to an overproduction of Reactive Oxygen Species (ROS) and to an imbalance of redox homeostasis (Wang et al., 2018). ROS are produced in plastids in the forms of singlet oxygen, superoxide anion (O_2_-), hydroxyl radicals and hydrogen peroxide (H_2_O_2_) (Noctor et al., 2002). ROS accumulation is controlled by scavenging and antioxidant machinery, including enzymes such as superoxide dismutase (SOD), catalases (CAT), ascorbate peroxidases (APX), and low molecular weight metabolites, like ascorbate (ASC), glutathione (GSH), tocopherols and carotenoids (Foyer and Noctor, 2013; Das et al., 2015). Although ROS were initially recognized as toxic by-products, a large number of evidence has shown the important role that these molecules may have in many essential plant processes (Farnese et al., 2016; Mittler, 2017). The role of ROS as oxidants or components of redox signaling mostly depends on a fine balance between the production and scavenging of these molecules in different organelles (Mittler, 2017).

In response to stress conditions, ROS can leave their production sites and, acting as secondary messengers, activate several signaling events (Pogson et al., 2008; Suzuki et al., 2012; Sgobba et al., 2015). In response to high temperatures, for instance, ROS act as retrograde signals, transmitting to the nucleus the redox alterations occurring in plastids (Singh et al., 2015; Hu et al., 2020);. In particular, ROS have been observed to elicit and regulate antioxidant enzymes and Heat Shock Proteins (HSPs) (Nishizawa et al., 2006; Volkov et al., 2006; Dickinson et al., 2018). Moreover, the presence of heat shock elements (HSE) in the promoter region of the Arabidopsis *APX1* and *APX2*, together with the increased thermo-sensitivity of Arabidopsis mutants defective in the biosynthetic pathways of antioxidants, supports the idea that a tight connection between ROS homeostasis and acclimation to HS exists (Pnueli et al., 2003; Larkindale et al., 2005).

In the last decades, plastid-localized Genomes Uncoupled (GUN) proteins have been identified as crucial in several processes involved in retrograde signaling (Susek et al., 1993; Mochizuki et al., 2001; Larkin et al., 2003; Strand et al., 2003; Koussevitzky et al., 2007; Woodson et al., 2011). Through chemical alteration of chloroplast biogenesis and physiology by either lincomycin (Lin) or norflurazon (NF) treatments, respectively, six *gun* mutants were isolated (Susek et al., 1993). After exposure to NF, all *gun* mutants expressed photosynthesis-associated nuclear genes (PhANGs), which on the contrary were repressed in wild type seedlings. Thus, it has been assumed that mutations in *GUN* genes led to the uncoupling of nuclear gene expression (NGE) with respect to the functional state of the chloroplast (Nott et al., 2006). GUN1 is a nuclear-encoded pentatricopeptide repeat protein with a C-terminal Small MutS-Relate domain, described as key player of plastid-to-nucleus retrograde signaling, response and adaptation to environmental challenges and plastid development (Koussevitzky et al., 2007; Wu et al., 2018; Pesaresi and Kim, 2019). Based on its amino acid sequence, GUN1 was initially identified as a nucleic acid-binding protein involved in DNA metabolism, gene expression, and DNA repair in the plastids (Koussevitzky et al., 2007). Successively, it has been proposed that GUN1 interacts with proteins rather than with nucleic acids. Among GUN1-interacting proteins, enzymes of the tetrapyrrole biosynthesis pathway and several proteins that participate in plastid gene expression (PGE) and protein homeostasis, such as plastid chaperons, have been identified (Colombo et al., 2016; Tadini et al., 2016; Zhao et al., 2019; Tadini et al., 2020; Wu and Bock, 2021). The identification of GUN1 putative interactors highlighted the role of GUN1 as a hub of multiple retrograde signaling pathways.

Despite the great attention on GUN1 role in the communication between chloroplast and nucleus, little information exists about its involvement in the signaling defense activated in response to HS. Here, we studied the role and interplay of GUN1 and redox signaling in heat stress response (HSR). The results indicate that *gun1* mutants are more sensitive to HS than wild-type plants and suggest that GUN1 could be required for basal thermotolerance, participating in the ROS-dependent oxidization of cellular environment, which is the basis for communication of plastid impairment to the nucleus.

## Materials and methods

### Plant materials, growth conditions and heat stress treatment

The Arabidopsis (*Arabidopsis thaliana*, genetic background Col-0) *gun1-102* T-DNA insertion mutant was previously described in Tadini et al. (2016). Wild type (wt) and *gun1-102* (hereafter indicated as *gun1*) seeds were surface-sterilized and sown out on Murashige and Skoog medium (Duchefa, Haarlem, The Netherlands) supplemented with 2% (w/v) sucrose and 1.5% (w/v) Phyto-Agar (Duchefa). After 2 days of stratification at 4°C in the dark, plantlets were grown in a growth chamber for 15 days (22°C, 80 μmol m^−2^ sec^−1^ on 16 h/8 h light/dark cycles).

On day 15, Arabidopsis wild-type and *gun1* plants were exposed to heat stress (45°C for 2 hours) according to Ling et al. (2018). To allow short-term and long-term physiological recovery, plants were then incubated in growth conditions (22°C) for 3 hours or 2 days, respectively. Samples for analysis were collected before HS (C), immediately after HS treatment, and after 3 hours (R) and 2 days (2d-RHS) of physiological recovery. Control plants for the experiments of 2d-RHS were collected after 17 days of growth at 22°C. Each biological replicate consisted of 90 plantlets per condition. Five biological replicates per timepoint were used while each experiment was repeated at least three times.

To measure root length in control, HS and recovery conditions, agar plates were oriented vertically in comparable growth conditions described above. To determine pigment contents leaves were separated from the roots, frozen in liquid nitrogen and stored at -80°C until analysis.

### Determination of pigment content and maximum quantum yield of PSII

For pigment quantification, leaf samples (50 mg) were ground in liquid nitrogen with 80% acetone (1:20 w/v) and the homogenates centrifuged at 20,000 g for 20 minutes at 4°C. The supernatant absorbances at 663.2, 646.8 and 470 nm were spectrophotometrically measured according to Zhang and Kirkham (1996). Content of chlorophyll a (Chl a) and chlorophyll b (Chl b), as well as total carotenoids (xanthophyll and β-carotene), expressed as μg g^-1^ fresh weight, were calculated according to Lichtenthaler (1987):


$$\text{Chlorophyll a }=\;12.25{\text{A}}_{663.2}\;-\;2.79{\text{A}}_{646.8}$$

$$\text{Ch;prophyll b b}=21.50{\text{A}}_{646.8}-5.10{\text{A}}_{663.2}$$

$$\text{Carotenoids }=\;\left(1000{\text{A}}_{470}-\;1.82{\text{C}}_{\text{a}}–\;85.02{\text{C}}_{\text{b}}\right)/198$$

The maximum quantum yield of PSII (*Fv/Fm*) was measured by using the Imaging PAM (Walz, Effeltrich, Germany) as described in Tadini et al. (2012).

### Proteasome activity

Proteasome activity was determined spectrofluorometrically by using the fluorogenic substrate Suc-LLYY-NH-AMC (Calbiochem), according to Paradiso et al. (2020). Arabidopsis plantlets were ground in liquid nitrogen and homogenized in a 1:3 (w/v) ratio with extraction buffer (50 mM Hepes-KOH, pH 7.2, 2 mM DTT, 2 mM ATP, 250 mM sucrose). After centrifugation at 20,000xg for 15 min at 4°C, supernatants were collected. 660 µL of samples, with 1mg mL^-1^ protein concentration, were mixed with 40 µL of assay buffer (100 mM Hepes-KOH, pH 7.8, 5mM MgCl_2_, 10 mM KCl, 2 mM ATP). After 15 min of incubation at 30°C in the dark, the reaction was started by the addition of the fluorogenic substrate. The release of amino-methyl-coumarin (360 nm ex/460 nm em) was monitored between 0 and 120 min by RF-6000 spectrofluorophotometer (Shimadzu Corporation, Japan). Protein concentration was measured using Protein Assay System (Bio-Rad, Hercules, CA, USA) according to Bradford (1976), with serum albumin as standard.

### Determination of ROS and oxidative markers

*In situ* O_2_- and H_2_O_2_ accumulation in leaves was detected with nitroblue tetrazolium (NBT) and 3,3-diaminobenzidine (DAB), respectively, as described in Fortunato et al. (2022). The staining intensity was digitally acquired and quantified by ImageJ software (https://imagej.nih.gov/ij/). The relative O_2_- and H_2_O_2_ levels were calculated as the percentage of NBT- and DAB-stained area of leaves, respectively.

The level of lipid peroxidation was evaluated in terms of malondialdehyde (MDA) content determined by the TBA reaction, as described by Paradiso et al. (2008). The amount of MDA-TBA complex was calculated using an extinction coefficient of 155 mM^-1^ cm^-1^.

Protein oxidation was spectrophotometrically determined by measuring the content of carbonyl-groups reacting with dinitrophenylhydrazine (DNPH), according to Romero-Puertas et al. (2002). Carbonyl content was calculated using an extinction coefficient of 22 mM^-1^ cm^-1^.

### Analysis of enzymatic and non-enzymatic antioxidants

For ascorbate (ASC) and glutathione (GSH) analysis, 0.3 g of samples were homogenized at 4°C with 1.8 mL 5% (v/v) trichloroacetic acid. After centrifugation at 18,000 x g for 20 minutes, the supernatants were collected and ASC and GSH levels were determined through the colorimetric assay described in de Pinto et al. (1999).

For quantifying the enzymatic antioxidant activities, 100 mg of samples were ground to fine powder in liquid nitrogen and mixed with 0.4 mL extraction buffer containing 50mM Tris-HCl pH 7.5, 0.05% (w/v) cysteine, 0.1% bovine serum albumin, 1 mM phenylmethanesulfonylfluoride. To determine the ascorbate peroxidase activity, 1 mM ASC was added to the buffer. After centrifugation at 20,000 x g for 20 minutes at 4°C, the supernatants were used for the spectrophotometric analysis.

Superoxide dismutase (SOD, EC 1.15.1.1) and catalase (CAT, EC 1.11.1.6) activities were spectrophotometrically determined following the methods described in Paradiso et al. (2020). Ascorbate peroxidase (APX, EC 1.11.1.11) was assayed according to de Pinto et al. (2000).

For Western Blot analyses of SOD, CAT and APX, total proteins were extracted from plantlets as described by Fortunato et al. (2022) and successively separated by SDS PAGE. Then, proteins were electrophoretically transferred to polyvinylidene fluoride membranes and incubated with the following specific antibodies: L-ascorbate peroxidase primary polyclonal antibody (n. AS08 368, Agrisera Vännäs, Sweden), which recognizes thylakoidal, stromal and cytosolic isoforms; Catalase (peroxisomal marker) primary polyclonal antibody (n. AS09 501, Agrisera Vännäs, Sweden); Fe-SOD primary polyclonal antibody (n. AS06 125, Agrisera Vännäs, Sweden), and the FTSH5 kindly donated by Wataru Sakamoto (Okayama University). As secondary antibody, the horseradish peroxidase (HRP)- conjugate Anti-Rabbit IgG (Promega, Madison, WI, USA) was used. Chemiluminescent signals were detected and quantified by ChemiDoc MP Imaging System and Quantity One^®^ software (Bio-Rad Laboratories, Hercules, CA, USA), respectively.

### Quantitative real-time PCR

Total RNA extraction was achieved using the Nippon Genetics Kit according to manufacturer protocol, using 50 mg (fresh weight) of leaf material. RNA concentration was determined by measuring the absorbance at 260 nm with NanoDrop2000 (Thermo Fisher Scientific, USA). cDNA was synthesized from 2μg of total RNA, using the iScript™ cDNA Synthesis Kit purchased by Bio-Rad according to the manufacturer’s instructions. Gene expression analysis (qPCR) was performed using the BIO-RAD CFX Connect system (Bio-Rad, Hercules, CA, USA) employing 37.5 ng of cDNA for each reaction and SsoAdvanced Universal SYBR Green Supermix (Bio-Rad), according to the manufacturer’s instruction for the detection system (Bio-Rad). *Ubiquitin10* (At4g05320) and *Actin8* (At1g49240) were used as housekeeping genes and three technical replicates were performed for each biological replicate (n=3). In all experiments, no template controls were also used. Housekeeping data were normalized according to Riedel et al. (2014).

Primers for quantitative real-time PCR (qRT-PCR) were designed by using Primer3 software (http://primer3.ut.ee/) and then double-checked using net primer software (http://www.premierbiosoft.com/netprimer/), except for the housekeeper primers (Giuntoli et al., 2017). Primer sequences used for quantitative PCR (qPCR) analyses are reported in **Table S1**.

Separation of real-time PCR products on 2% (w/v) agarose gels revealed single bands of the expected molecular weight. Relative quantification was performed according to the comparative Ct (threshold cycle) method (2^−ΔΔCt^); (Livak and Schmittgen, 2001).

### Statistical analysis

The data were expressed as the means ± standard error (SE). One-way analysis of variance (ANOVA) followed by a *post-hoc* Tukey’s comparison test was used to calculate the difference between genotypes and treatments. Differences were considered statistically significant at a p-value< 0.05. All statistical analyses were performed by Minitab software (Minitab Inc., State College, PA, USA).

## Results

### Heat stress sensitivity of wild type and *gun1* plantlets

**Figure 1:**
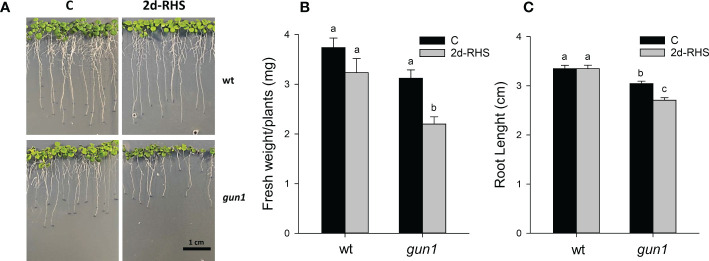
Growth parameters of 17-day-old wt and *gun1* plantlets, grown at control temperature **(C)** or subjected, after 15 days, to Heat Stress (HS; 2 hours at 45°C), followed by long-term recovery (2d-RHS; 2 days at 22°C). **(A)** Representative image of visible phenotypes, **(B)** Fresh Weight and **(C)** Root Length of plantlets measurements. The values are the means ± standard errors of five independent experiments. Different letters indicate significant differences obtained by one-way ANOVA test (P< 0.05).

**Figure 2:**
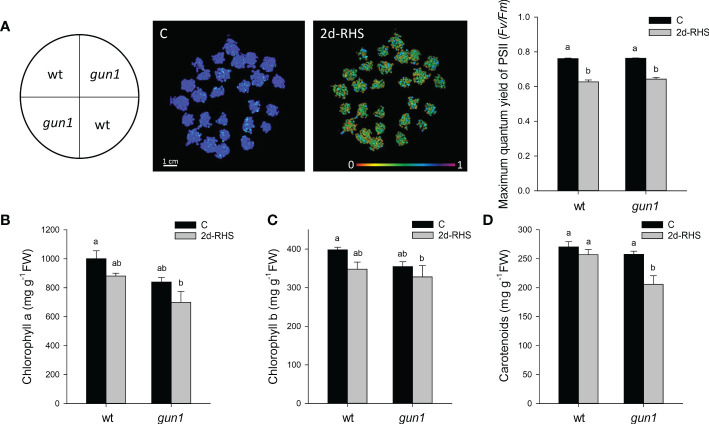
Photosynthetic parameters of 17 days-old wt and *gun1* plantlets, grown at control temperature **(C)** or subjected, after 15 days of growth, to Heat Stress (HS; 2 hours at 45°C), followed by long-term recovery (2d-RHS; 2 days at 22°C). **(A)** Photosynthetic performance of wt and *gun1*. The maximum quantum yield of PSII (*Fv/Fm*) was measured by using the IMAGING PAM (WALZ). **(B)** Chlorophyll a, **(C)** chlorophyll b and **(D)** carotenoids contents were as well determined. The values are the means ± standard errors of five independent experiments. Different letters indicate significant differences obtained by one-way ANOVA test (P< 0.05).

To analyze heat stress sensitivity, growth rate parameters were measured in 17-day-old wild type (wt) and *gun1* mutant plantlets. Plants were grown for 15 days at 22°C and exposed for 2 hours at 45°C (Heat Stress, HS). Plantlets were then incubated transferred in growth conditions to their optimal growth conditions (22°C; see *Materials and methods*) for 2 days to allow physiological recovery (2d-RHS), which was assessed by monitoring the photosynthetic parameter Fv/Fm. As a control (C), wt and *gun1* plants were grown at 22°C for 17 days. The phenotypical analysis showed that, after 2 days of recovery from HS (2d-RHS), *gun1* plantlets were significantly smaller than wt (**Figure 1A**). The visible phenotype was confirmed by measuring whole plant fresh weight, which resulted significantly decreased in *gun1* plantlets subjected to HS but did not show significant differences in wt plantlets, when compared to the untreated controls (**Figure 1B**). Root length did not change in HS-treated wt while, on the contrary, *gun1* roots were shorter than wt, already under control conditions, and the exposure to HS further reduced root elongation (**Figure 1C**). The photosynthetic efficiency, measured as the maximum quantum yield of PSII (*Fv/Fm*), resulted decreased in a similar way in wt and *gun1* plantlets at 2d-RHS, when compared to control conditions (**Figure 2A**). Consistently, chlorophyll a and b content did not change significantly between wt and *gun1* (**Figures 2B, C**). On the other hand, a significant drop in carotenoid accumulation occurred in *gun1* mutant only (**Figure 2D**).

**Figure 3:**
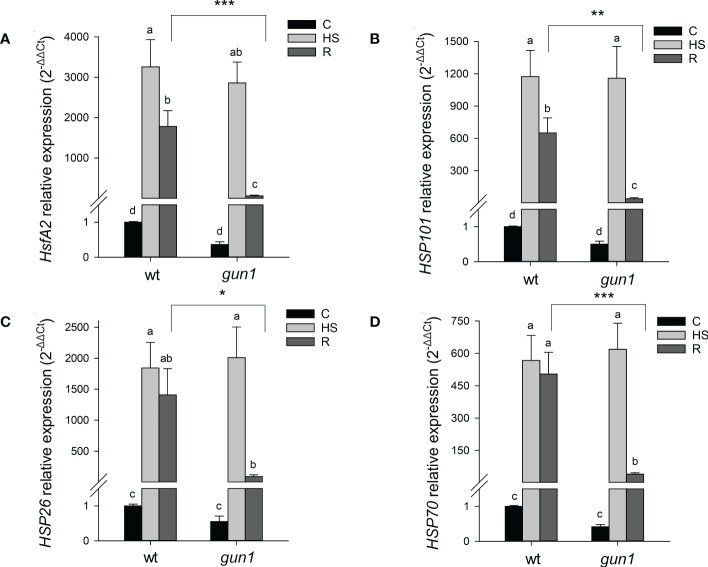
Relative expression of **(A)** *HsfA2*, **(B)** *HSP101*, **(C)** *HSP26* and **(D)** *HSP70* in 15 days-old wt and *gun1* plantlets, grown at control temperature **(C)** or subjected to Heat Stress (HS; 2 hours at 45°C), followed by short recovery (R; 3 hours at 22°C). The expression level of *HsfA2* and *HSPs* was normalized to that of *Ubiquitin10* (At4g05320) and *Actin8* (At1g49240) as internal references. For each sample, gene expression was related to the wt in control conditions **(C)**, set as 1. The values are the means ± SEs from three independent experiments, with three technical replicates for each experiment. Different letters indicate significant differences obtained by one-way ANOVA test (p< 0.05). T-test was applied to compare R samples among the genotypes. *p ≤ 0.05; **p ≤ 0.01; ***p ≤ 0.001.

To dissect more in detail the molecular mechanisms underlying *gun1* sensitivity to heat, the transcript level of heat-dependent genes was assessed by quantitative Real-Time PCR (qRT-PCR) in 15 days-old plants before (C), right after the heat stress (HS, 2 hours at 45°C) and upon 3 hours of recovery at 22°C (R), when phenotypic differences were not detectable (**Table S2**). To this aim, the expression level of heat shock factor A2 (*HsfA2*), a key regulator of the heat stress response, and some heat shock proteins (*HSPs*), was studied. A significant and similar increase in the transcript levels of the nuclear *HsfA2*, the cytosolic *HSP101* and *HSP70* and the chloroplast *HSP26* occurred in response to HS in both wt and *gun1* genotypes. After 3h recovery (R), *HsfA2* and *HSP101* expression decreased in both genotypes, however, the reduction of both transcripts was more marked in *gun1* than in wt (**Figures 3A, B**). In addition, the expression of *HSP70* and *HSP26* did not change significantly after recovery (R) in wt, unlike in *gun1* (**Figures 3C, D**). To verify whether in *gun1* mutants heat stress could induce cytosolic folding stress, caused by the accumulation of plastid protein precursors and over-accumulation of cytosolic HSPs, as occurred when the mutants were grown in lincomycin conditions (Wu et al., 2019; Tadini et al., 2020), proteasomal activity and accumulation of FTSH5 plastid protease were analyzed. The proteasome activity in wt and *gun1* plantlets grown in control conditions, upon HS and after recovery did not display significant differences (**Figure S1**). Moreover, the accumulation of FTSH5 plastid protease pre-protein was not detectable upon HS treatment, while resulted to be accumulating in Lin-treated *gun1* seedlings, suggesting that Lin and HS trigger different non-overlapping signaling mechanisms (**Figure S1**).

### ROS accumulation, oxidative markers, and hydrophilic antioxidants in wild type and *gun1* plantlets during HSR

**Figure 4:**
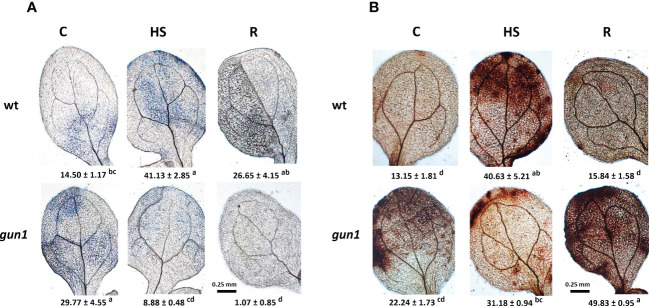
Accumulation of superoxide anion (O_2_^−^) and hydrogen peroxide (H_2_O_2_) in 15 days-old wt and *gun1* plantlets, grown at control temperature **(C)** or subjected to Heat Stress (HS; 2 hours at 45°C), followed by short recovery (R; 3 hours at 22°C). Representative images of **(A)** O_2_^−^ accumulation, visualized by nitroblue tetrazolium (NBT)-staining and **(B)** H_2_O_2_ accumulation, visualized by diaminobenzidine (DAB)-staining. O_2_^−^ and H_2_O_2_ analyses were repeated three times showing reproducible results. The percentage area ( ± SE) of 60 leaves (20 for each experiment) stained with NBT and DAB, respectively, are indicated. Letters indicate significant differences obtained by one-way ANOVA test (P< 0.05).

**Figure 5:**
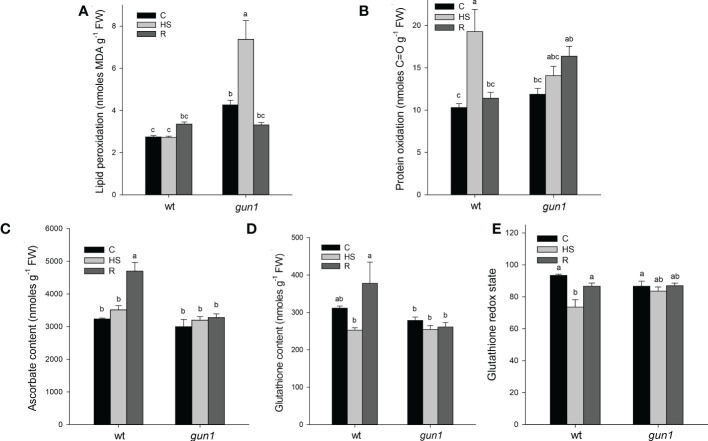
Oxidative markers and non-enzymatic antioxidants of Arabidopsis wild type (wt) and *gun1* plantlets, grown at control temperature **(C)** or subjected to Heat Stress (HS; 2 hours at 45°C), followed by short recovery (R; 3 hours at 22°C). **(A)** Lipid peroxidation, measured as malondialdehyde content and **(B)** protein oxidation measured as total protein carbonyl groups. **(C)** Total ascorbate and **(D)** glutathione contents. **(E)** Glutathione redox state calculated as percentage of the ratio between reduced and total glutathione. The values are the means ± standard errors of five independent experiments. Different letters indicate significant differences obtained by one-way ANOVA test (P< 0.05).

ROS accumulation in response to HS was different between control and mutant genotypes (**Figure 4**). Under control conditions, the level of O_2_-, visualized by NBT-staining, was significantly higher in *gun1* leaf tissue than in wt (**Figure 4A**). Nevertheless, in the *gun1* genetic background, the accumulation of O_2_- decreased after HS, reaching bottom values after recovery (R). On the other hand, in wt, HS caused a prompt accumulation of O_2_-, which successively decreased during the R phase (**Figure 4A**). Similarly, in wt, H_2_O_2_ levels, visualized by DAB-staining, increased after HS and returned to values comparable with control during the recovery (R) (**Figure 4B**). On the contrary, in *gun1*, H_2_O_2_ levels did not vary significantly after HS, but showed a high accumulation after recovery (**Figure 4B**). Furthermore, the level of lipid peroxidation was higher in *gun1* than in wt in control conditions (**Figure 5A**). This oxidative marker did not vary significantly in response to HS in wt plantlets, whereas it transiently increased in *gun1* mutants, to return to a baseline level after recovery. In wt, protein oxidation increased after HS and returned to values comparable with the control after recovery while, in *gun1* mutant background, the total level of protein carbonyl groups did not show significant changes after HS and R (**Figure 5B**). The total content of two major hydrophilic antioxidants, ascorbate (ASC) and glutathione (GSH), did not vary significantly between wt and *gun1*, under control conditions (**Figures 5C, D**). In *gun1*, the total content of the two antioxidants did not change either upon HS or after recovery (R). On the other hand, in wt total glutathione levels were lower after HS and both the antioxidants increased after recovery (**Figures 5C, D**). Moreover, only in wild type seedlings HS reduced the glutathione redox state, which returned to values comparable to control after recovery (**Figure 5E**).

### Behaviour of ROS scavenging enzymes in wild type and *gun1* plantlets upon heat stress

To clarify the different accumulation of ROS in the two genotypes during the HSR, the behavior of the main ROS scavenging enzymes, namely superoxide dismutase (SOD), catalase (CAT) and ascorbate peroxidase (APX), was investigated.

**Figure 6:**
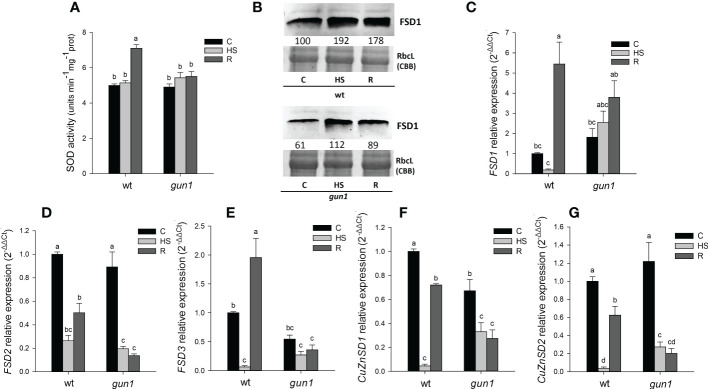
Superoxide dismutase (SOD) behavior in 15 days-old wt and *gun1* plantlets, grown at control temperature **(C)** or subjected to Heat Stress (HS; 2 hours at 45°C), followed by short recovery (R; 3 hours at 22°C). **(A)** Total SOD activity; values are the means ± standard errors (SE) of five independent experiments. Letters indicate significant differences obtained by one-way ANOVA test (P< 0.05). **(B)** Representative images from three independent experiments of FSD1 immuno-blotting; each well was loaded with 30 μg of proteins. Coomassie Brilliant Blue (CBB) staining of the gel served as a loading control. Quantification of signals (by Quantity One^®^) relative to the wt in control conditions (100%) is provided below the panel. Relative expression of **(C)** *FSD1*, **(D)** *FSD2*, **(E)** *FSD3*, **(F)** *CuZnSD1* **(G)** and *CuZnSD2*. The expression level of *SOD* genes was normalized to that of *Ubiquitin10* (At4g05320) and *Actin8* (At1g49240) as internal references. For each sample, gene expression was related to the wt in control conditions **(C)**. Values are expressed as means ± SE from three independent experiments, with three technical replicates for each experiment. Different letters indicate significant differences obtained by one-way ANOVA test (p< 0.05).

Total SOD activity was similar in wt and *gun1* under control conditions and did not change significantly upon HS in both genotypes. After recovery (R), a rise in SOD activity occurred in wt control only (**Figure 6A**). The levels of FSD1 protein and transcript were analyzed by immunoblotting and qRT-PCR, respectively (**Figures 6B, C**). FSD1 protein accumulation was higher in wt than in *gun1* under control conditions. In both genotypes the protein level increased in response to HS, remaining at a higher level than control also after recovery (R) (**Figure 6B**). In wt plantlets, after HS a decrease in *FSD1* expression occurred, while during the recovery a clear and significant increase in the transcript level was observed (**Figure 6C**). The two thylakoidal Fe-SOD, *FSD2* and *FSD3* (Myouga et al., 2008), behaved differently when compared to *FSD1* (**Figures 6D, E**), which besides being present in the stroma of the chloroplast is also localized in the cytoplasm and nuclei (Dvořák et al., 2021). In both the genotypes, HS caused a strong decrease in *FSD2* expression, which remained low in *gun1* and increased in wt after recovery (**Figure 6D**). On the other hand, *FSD3* expression did not change in *gun1* in response to HS, while in wt decreased after HS and increased after recovery (**Figure 6E**). HS reduced the expression of cytosolic and chloroplastic copper/zinc superoxide dismutases (*CuZnSD1* and *CuZnSD2*, respectively) in both genotypes. However, after recovery (R), the transcript level of *CuZnSD1* and *CuZnSD2* was partially restored only in wt (**Figures 6F, G**).

**Figure 7:**
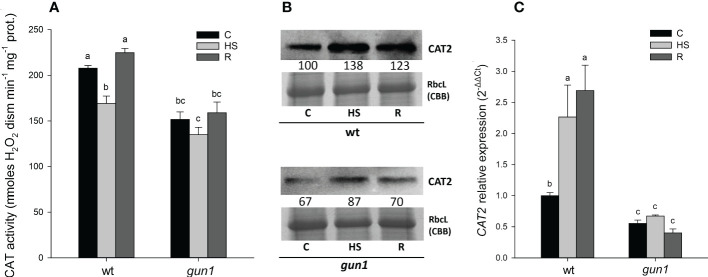
Catalase (CAT) behavior in 15 days-old wt and *gun1* plantlets, grown at control temperature **(C)** or subjected to Heat Stress (HS; 2 hours at 45°C), followed by short recovery (R; 3 hours at 22°C). **(A)** Total CAT activity. Values are the means ± standard errors (SE) of five independent experiments. Letters indicate significant differences obtained by one-way ANOVA test (P< 0.05). **(B)** Representative image from three independent experiments of CAT2 immunoblotting, each well was loaded with 30 μg of proteins. CBB staining of the gel is shown as equal loading control. Quantification of signals (by Quantity One^®^) relative to the wt in control conditions (100%) is provided below the panel. **(C)** Relative expression of *CAT2*. The expression level of *CAT2* was normalized to that of *Ubiquitin 10* (At4g05320) and *Actin8* (At1g49240) as internal references. For each sample, gene expression was related to the wt in control conditions **(C).** Values are the means ± SE from three independent experiments, with three technical replicates for each experiment. Letters indicate significant differences obtained by one-way ANOVA test (p< 0.05).

In control conditions, total CAT activity, together with CAT2 protein and transcript levels were lower in *gun1* than in wt (**Figure 7**). HS caused, however, transient inhibition of CAT activity in wt only (**Figure 7A**). Despite the decrease in CAT activity observed in wt samples, CAT2 protein and transcript increased after HS, while did not significantly change in *gun1* (**Figures 7B, C**).

**Figure 8:**
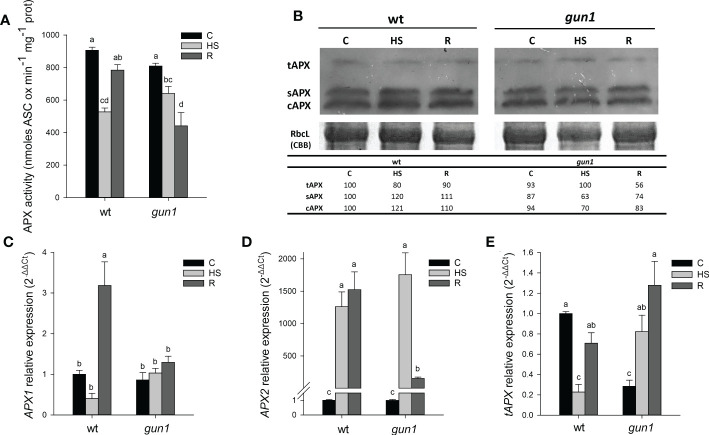
Ascorbate peroxidase (APX) behavior in 15 days-old wt and *gun1* plantlets, grown at control temperature **(C)** or subjected to Heat Stress (HS; 2 hours at 45°C), followed by short recovery (R; 3 hours at 22°C). **(A)** Total APX activity; values indicate means ± standard errors (SE) of five independent experiments. Letters indicate significant differences obtained by one-way ANOVA test (P< 0.05). **(B)** Representative image from three independent experiments of APX immuno-blotting; each well was loaded with 30 μg of proteins. CBB staining of the gel served as a loading control. Quantification of signals (by Quantity One^®^) relative to the wt in control conditions (100%) is provided below the panel tAPX, sAPX and cAPX are thylakoidal, stromal and cytosolic APX, respectively. Relative expression of **(C)** *APX1*, **(D)** *APX2* and **(E)** *tAPX*. The expression level of APX genes was normalized to that of *Ubiquitin10* (At4g05320) and *Actin8* (At1g49240) as internal references. For each sample, gene expression was related to wt in control conditions **(C)**. Values indicate means ± SE from three independent experiments, with three technical replicates for each experiment. Different letters indicate significant differences obtained by one-way ANOVA test (p< 0.05).

Moreover, after HS, total APX activity decreased in both genotypes, with a greater intensity in wt than in *gun1*. However, after recovery, APX activity was restored to control (C) level in wt while further decreased in *gun1* (**Figure 8A**). Western blotting analysis showed that in wt, the accumulation of cytosolic and stromal APX was slightly increased upon HS, while the decrease of thylakoidal isoform was observed. On the other hand, in *gun1* samples, cytosolic and stromal APX isoenzymes showed a progressive decrease while tAPX accumulated in response to HS and decreased in R (**Figure 8B**).

In wt, the expression of cytosolic *APX1* was strongly reduced after HS and significantly increased after recovery, while in *gun1* not significant changes occurred (**Figure 8C**). The HS-inducible APX2 showed, however, highly increased expression after HS in both the genotypes. After recovery (R), *APX2* transcript remained high in wt and partially decreased in *gun1* (**Figure 8C**). At last, the expression of *tAPX* under control conditions was significantly lower in *gun1* than in wt. However, after HS a drop in *tAPX* transcript occurred in wt, while a progressive increase after HS and in R was observed in *gun1* mutants (**Figure 8D**).

## Discussion

Retrograde signaling pathways allow the information flux from plastids to the nucleus. This intra-cellular communication becomes critical during chloroplast biogenesis (biogenic control) and upon alteration of plastid homeostasis in response to environmental stimuli (operational signaling) (Chan et al., 2016). GUN1-dependent signaling has been proposed as one of the main retrograde signaling pathways active during chloroplast biogenesis (Tadini et al., 2020; Shimizu and Masuda, 2021; Wu and Bock, 2021). Nevertheless, multiple evidence suggests that GUN1 also operates in adult plants, contributing to the operational control of chloroplasts (Cheng et al., 2011; Tadini et al., 2016; Guo et al., 2020). Indeed, GUN1 undergoes ta rapid turnover by the Clp protease, unless it becomes stable during the early stages of chloroplast biogenesis, and under stress conditions that trigger retrograde signaling pathways (Wu et al., 2018; Pesaresi and Kim, 2019).

GUN1 has been reported to be required for cold acclimation, as *gun1* seedlings fail to develop green functional chloroplasts when grown at 4°C (Marino et al., 2019). Moreover, the involvement of GUN1 in response to HS has been previously indicated by showing that *gun1* mutants have reduced basal thermotolerance but do not appear to be impaired in acquired thermotolerance (Miller et al., 2007). In accordance, our data indicate the *gun1* mutants are more sensitive to HS than wt, as demonstrated by the reduced fresh weight and the inhibition of root elongation at 2 days of physiological recovery from HS (**Figure 1**). Furthermore, at 2d-RHS, despite *gun1* mutants show similar reduction in photosynthetic efficiency than wild type plants, have a reduced content of carotenoids (**Figure 2**). The decrease in carotenoids content may contribute to higher sensitivity to HS, since these molecules act not only as quenchers of triplet chlorophyll and singlet oxygen but might also stabilize and photo-protect the lipid phase of the thylakoid membranes (Havaux, 1998).

The lowered heat tolerance of *gun1* mutants is not due to cytosolic folding stress, which instead occurs in response lincomycin treatment (**Figure S1**; Wu et al., 2019; Tadini et al., 2020), suggesting the involvement of a different signaling mechanism. Moreover, the reduced basal thermotolerance of *gun1* mutants cannot be explained by the failure in the induction of *HSPs*, since in *gun1*, *HsfA2* and the cytosolic and chloroplastic *HSPs* analyzed were highly expressed after HS as in wild type plants. However, the higher decrease of *HSPs* after 3 hours of recovery from HS corroborates the idea of a lower thermotolerance of *gun1* mutants compared to wild type plants (**Figure 3**; Ahn et al., 2004; Charng et al., 2007).

It has been recently reported that during biogenic retrograde signaling, GUN1 mediates the formation of an H_2_O_2_- dependent oxidized environment, which might represent a redox-mediated communication pathway, aimed to signal the perturbation of chloroplast development (Fortunato et al., 2022).

A plethora of literature data indicate that environmental stresses, including high temperatures, lead to oxidative bursts of O_2_- and/or H_2_O_2_ in plants (Foyer et al., 1997; Dat et al., 1998; Vallélian-Bindschedler et al., 1998). Accordingly, ROS produced in chloroplasts can work as plastid signals to activate the expression of genes coding for antioxidant enzymes and to fine-tune the stress-responsive apparatus for more effective adaptation to stresses (Sun and Guo, 2016). Chloroplasts have been shown to play an important role in heat-induced ROS accumulation and the subsequent expression of nuclear heat-responsive genes (Hu et al., 2020). The chloroplast-produced H_2_O_2_ working as signaling molecule for the heat-associated gene expression has been proposed as an interesting model for the generation of diurnal patterns of thermotolerance (Dickinson et al., 2018).

Our results show that in wt, immediately after HS, O_2_- and H_2_O_2_ values increase, returning to values comparable to control conditions after 3 hours of physiological recovery, while a transient increase in protein oxidation was observed (**Figures 4**, **5B**). This suggests that in this context ROS may contribute to oxidizing the cellular environment temporarily, triggering a signaling cascade. The transient oxidation of cellular environment has been confirmed by the changes in the glutathione redox state, which decreases after HS and returns to values comparable to control during the physiological recovery (**Figure 5D**). These results are in accordance with recent studies in which the redox-sensitive green fluorescent protein (roGFP2) was used to show that HS leads to increased oxidation in both cytosol and nucleus compartments. By analyzing transcript profiles of control and heat-stressed plantlets, the authors suggest that heat-induced changes in the nuclear redox state are essential for genetic and epigenetic regulation of HSR (Babbar et al., 2021).

In wt, the transient oxidative burst is also due to the lowered total activity of APX and CAT occurring immediately after HS (**Figures 7A, B).** Analyzing the protein levels of different APX isoenzymes, it should be noted that, despite the significant decrease of the total activity, the levels of cytosolic and stromal APX proteins increased. At least for the cytosolic APX, two observations could explain this apparent inconsistency: 1) Immediately after HS, the expression level of *APX2* transcript significantly increased, as expected (**Figure 8B**; Panchuk et al., 2002; Suzuki et al., 2013; Balfagón et al., 2018); 2) It has been reported that after HS, APX1 protein forms high molecular weight complexes, loses the H_2_O_2_ removal activity, and behaves as chaperone protein. Interestingly, when plants are recovered under physiological conditions, the APX protein returns to dimeric or oligomeric form, recovering its H_2_O_2_- removal activity required to prevent oxidative damage (Kaur et al., 2021). On the other hand, protein and transcript levels of thylakoidal APX decreased immediately after HS. In this case, the loss or inactivation of tAPX may function as a part of plastid to nucleus retrograde signaling as occurs in light-induced photooxidative stress (Maruta et al., 2012).

Interestingly, also the decrease in CAT activity did not overlap with protein and transcript levels of CAT2, which accumulate immediately after HS. CAT is a peroxisomal enzyme with a pivotal role in redox regulation (Mhamdi et al., 2012). It has been shown that CAT can physically interact with cytosolic stress signaling proteins in plants (Foyer et al., 2020). Thus, it is likely that, upon HS, CAT becomes restrained to the cytosol and mediates redox signaling, as it occurs in mammalians (Walton et al., 2017).

In wt, 3 hours after physiological recovery from HS, both non-enzymatic and enzymatic antioxidants significantly increased, lowering ROS accumulation and preventing oxidative damage (**Figures 4**–**8**). In particular, the increase in SOD activity is due to an increase in the expression level of almost all the isoenzymes analyzed and the recovery in APX activity is due to the increased protein and expression level of all APX isoforms (**Figure 8**).

It is interesting to note that *gun1* mutants grown under physiological conditions show a higher O_2_- accumulation and a greater level of lipid peroxidation than wt (**Figure 4A**), which suggests that *gun1* plastids are more inclined to suffer ROS-mediated damage (Ruckle et al., 2007; Fortunato et al., 2022). After HS, the decrease in O_2_- implies the formation of more reactive hydroxyl radicals, which promptly react with lipids, causing a further increase in lipid peroxidation (**Figure 5A**; Farmer and Mueller, 2013). However, unlike wt, *gun1* mutants fail to induce an oxidative burst immediately after HS, since no O_2_- neither H_2_O_2_ accumulate (**Figure 4**). Consistently, the content of antioxidants and the total activities of SOD and CAT did not show significant differences (**Figures 5**–**7**). The absence of a rise in H_2_O_2_ under HS may contribute to increased heat oxidative damage, as already suggested for *fsd2* and *fsd3* mutants (Bychkov et al., 2022).

In *gun1* mutants, after 3 hours of physiological recovery from HS non-enzymatic antioxidants, as well SOD and CAT activity do not significantly change, whereas a decline in total APX activity occurs, due to the failure in the rescue of the protein levels of chloroplastic and cytosolic APX. As a consequence, H_2_O_2_ accumulates, becoming responsible for oxidative damage. It has been reported that under photooxidative stress the absence of tAPX more than sAPX causes the accumulation of H_2_O_2_ and oxidized proteins (Maruta et al., 2010). Moreover, in the absence of the cytosolic APX1, the entire chloroplastic H_2_O_2_- scavenging system of Arabidopsis is impaired (Davletova et al., 2005), Thus, in *gun1* mutants the absence of the induction of APX1 expression (**Figure 8C**), could be in part responsible for the failure in thermotolerance acquisition.

However, in *gun1* mutants, the behavior of tAPX deserves more attention; indeed, it should be noted that the expression level of tAPX, which is lower than wt under physiological growth conditions, increased after HS and during the physiological recovery, despite the failure in the accumulation of the protein (**Figures 8B, E**). These results indicate that the expression of tAPX gene is under the control of the GUN1-mediated signaling pathway, albeit protein amount also appears to be subjected to post-transcriptional regulatory mechanisms that include cytosolic inhibition of protein translation and ubiquitin-mediated protein degradation (Wu et al., 2019; Tadini et al., 2020). This regulation has been described for several *PhANGs*-encoded proteins and, among those, tAPX itself (Wu et al., 2019).

## Conclusions

Our data suggest that the transient oxidative burst occurring after HS is mandatory in basal thermotolerance acquisition. Indeed, in wt plants, ROS and oxidation of the cellular environment function as signals to activate the expression of genes adjusting stress-responsive systems for more successful adaptation to HS.

After HS, *gun1* mutants fail to induce ROS accumulation promptly, impairing the proper HSR. This leads to accumulating ROS and oxidative damage during physiological recovery at growth temperature, resulting in enhanced sensitivity to HS.

The results support the idea that GUN1 is required to oxidize the cellular environment, participating in the acquisition of basal thermotolerance through the redox-dependent plastid-to-nucleus communication.

Our results also indicate a pivotal role of tAPX in GUN1-dependent HSR; further investigation will be aimed at clarifying the mechanisms involved in this signaling pathway.

## Data availability statement

The original contributions presented in the study are included in the article/**Supplementary Material**. Further inquiries can be directed to the corresponding author.

## Author contributions

CL, SF, FV, and MCdP conceived and designed research. CL, SF, ND, NJ, LT, and FV performed the experiments. MCdP advised on the experiments. CL, SF, LT, FV, and MCdP drafted the paper. MCdP and PP funded the project. All authors contributed to the discussion of the data and to the writing and agreed to the published version of the manuscript.
