# Supplementary material for: Chloroplast-localized GUN1 contributes to the acquisition of basal thermotolerance in Arabidopsis thaliana
Source: Front Plant Sci. 2022 Dec 22;13:1058831. doi: 10.3389/fpls.2022.1058831 (PMC9813751; doi:10.3389/fpls.2022.1058831)
Supplement: Supplementary file 1 [file DataSheet_1.pdf]

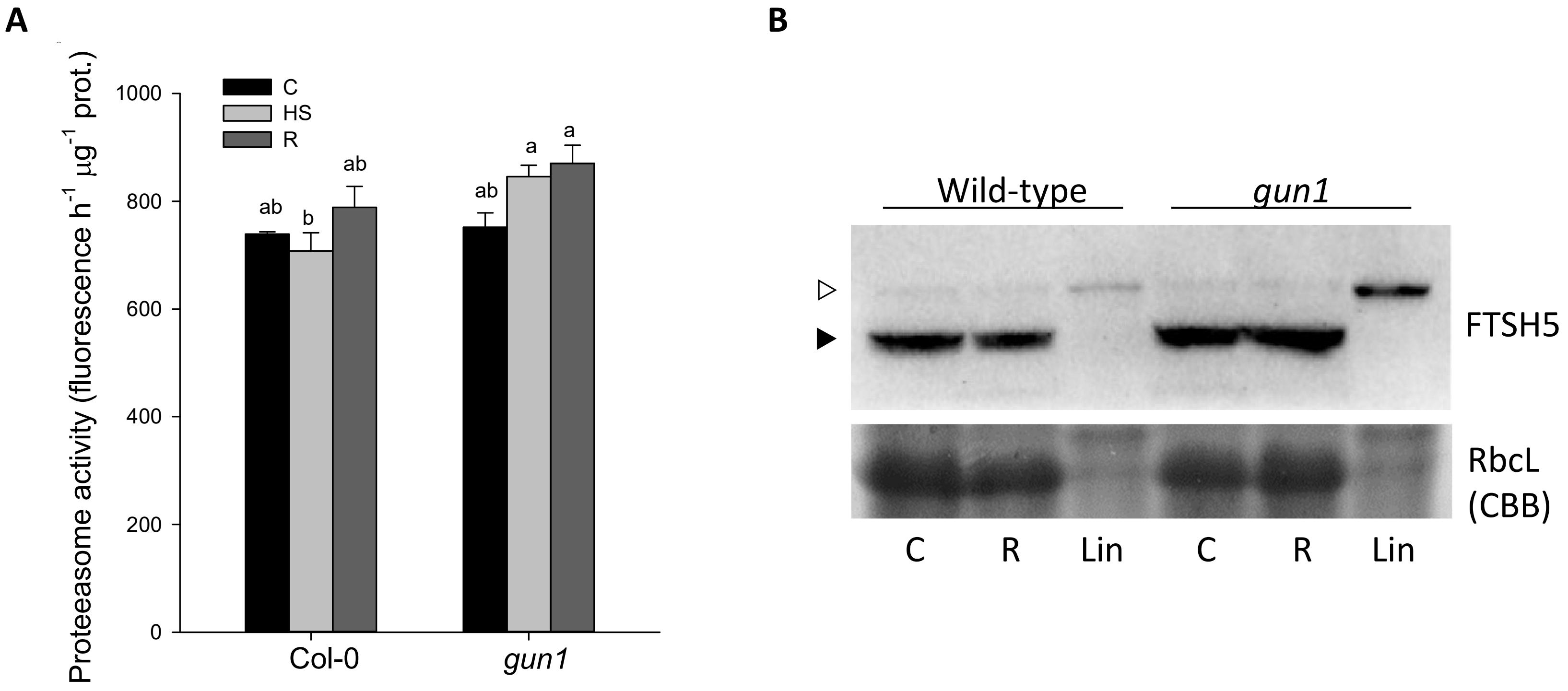

Figure S1 – Cytosolic folding stress evaluation in *gun1* mutant background. Measurements of proteasome activity (**A**). Immunoblot showing FTSH5 accumulation in wild-type and *gun1* genetic background grown in control condition (C), after 3 h recovery from heat shock (R) and in presence of 550 µM lincomycin (Lin) (**B**). Mature form and precursor bands are indicated by black or white arrowheads, respectively. The CBB-stained gel is shown as a loading control.
