# Supplementary material for: Chloroplast-localized GUN1 contributes to the acquisition of basal thermotolerance in Arabidopsis thaliana
Source: Front Plant Sci. 2022 Dec 22;13:1058831. doi: 10.3389/fpls.2022.1058831 (PMC9813751; doi:10.3389/fpls.2022.1058831)
Supplement: Supplementary file 2 [file Table_1.docx]

**Table S1.** List of primer pairs used for qPCR analysis of gene expression. The housekeeping genes are reported in bold.

| **Protein name** | **Gene name** | **AGI code** | **Forward primer** | **Reverse Primer** |
| --- | --- | --- | --- | --- |
| Heat Shock Protein 101 | ***HSP101*** | **At1g74310.1** | 5'-agggattccagtgacgagactt-3' | 5'-agggattccagtgacgagactt-3' |
| Heat Shock Transcription Factor A2 | ***HSFA2*** | **At2g26150.1** | 5'-ggcgttgaacaatccgaact-3' | 5'-ggcgttgaacaatccgaact-3' |
| Heat shock protein 26,5 | ***HSP26.5*** | **At1g52560.1** | 5'-cggctggtgaacaagaggat-3' | 5'-cggctggtgaacaagaggat-3' |
| Heat Shock Protein 70 | ***HSP70*** | **At3g12580.1** | 5'-gggcacgaacaaaggacaac-3' | 5'-gggcacgaacaaaggacaac-3' |
| Ascorbate Peroxidase 1 | ***APX1*** | **At1g07890.3** | 5'-cccgagagtcatggctgttt-3' | 5'-cccgagagtcatggctgttt-3' |
| Ascorbate Peroxidase 2 | ***APX2*** | **At3g09640.1** | 5'-atgtgtttggtcggatggga-3' | 5'-atgtgtttggtcggatggga-3' |
| Thylakoidal Ascorbate Peroxidase | ***tAPX*** | **At1g77490.1** | 5'-tcttggaagcgtcgcatctt-3' | 5'-tcttggaagcgtcgcatctt-3' |
| Fe Superoxide Dismutase 1 | ***FSD1*** | **At4g25100.1** | 5'-tcggctctttcccattgctt-3' | 5'-tcggctctttcccattgctt-3' |
| Catalase 2 | ***CAT2*** | **At4g35090.1** | 5'-ctgacatggtccacgctctt-3' | 5'-ctgacatggtccacgctctt-3' |
| Copper/Zinc Superoxide Dismutase 1 | ***CuZnSD1*** | **At1g08830.1** | 5'-ctgccaccttcacaatcactga-3' | 5'-ctgccaccttcacaatcactga-3' |
| Fe Superoxide Dismutase 2 | ***FSD2*** | **At5g51100.1** | 5'-gacaaaggtggctgtttccg-3' | 5'-gacaaaggtggctgtttccg-3' |
| Fe Superoxide Dismutase 3 | ***FSD3*** | **At5g23310.1** | 5'-acccaacatcccaatcgct-3' | 5'-acccaacatcccaatcgct-3' |
| Copper/Zinc Superoxide Dismutase 2 | ***CuZnSD2*** | **At2g28190.1** | 5'-acaacatgacacacggagct-3' | 5'-acaacatgacacacggagct-3' |
| **Polyubiquitin 10** | ***UBQ10*** | **At4g05320** | 5'-ggccttgtataatccctgatgaataag-3' | 5'-ggccttgtataatccctgatgaataag-3' |
| **Actin 8** | ***ACT8*** | **At1g49240** | 5'-gctttcctttgtcgctgtcg-3' | 5'-tcgtgatcacttgtccgtcg-3' |
