# Supplementary material for: Chloroplast-localized GUN1 contributes to the acquisition of basal thermotolerance in Arabidopsis thaliana
Source: Front Plant Sci. 2022 Dec 22;13:1058831. doi: 10.3389/fpls.2022.1058831 (PMC9813751; doi:10.3389/fpls.2022.1058831)
Supplement: Supplementary file 3 [file Table_2.docx]

**Table S2.** Fresh weight and root length measured in 15-days-old Col-0 and *gun1* seedlings, grown at control temperature (C) or subjected, after 15 days of growth, to Heat Stress (HS; 2 hours at 45°C), followed by recovery (R; 3 hours at 22°C). One-way analysis of variance (ANOVA) followed by a post-hoc Tukey’s comparison test was used to calculate the difference between genotypes and treatments. The values are the means ± Standard error of the mean (SEM) from five independent experiments, with three technical replicates for each experiment.

|  |  | **Fresh weight/plants (mg)** | | |  | **Root length (cm)** | | |
| --- | --- | --- | --- | --- | --- | --- | --- | --- |
| **Genotype** | **Treatment** | **Mean** | **SEM** | **Post-test results^a^** |  | **Mean** | **SEM** | **Post-test results** |
| **Col-0** | **C** | 3,45 | 0,16 | ns |  | 3,17 | 0,144 | ns |
|  | **HS** | 3,15 | 0,46 | ns |  | 3,32 | 0,114 | ns |
|  | **R** | 2,80 | 0,35 | ns |  | 3,34 | 0,137 | ns |
| ***gun1*** | **C** | 2,83 | 0,28 | ns |  | 2,68 | 0,166 | ns |
|  | **HS** | 2,48 | 0,56 | ns |  | 2,83 | 0,166 | ns |
|  | **R** | 2,59 | 0,28 | ns |  | 3,00 | 0,215 | ns |

^a^ns = not significant
